# Farnesoid X Receptor Activation Stimulates Organic Cations Transport in Human Renal Proximal Tubular Cells

**DOI:** 10.3390/ijms21176078

**Published:** 2020-08-24

**Authors:** Teerasak Wongwan, Varanuj Chatsudthipong, Sunhapas Soodvilai

**Affiliations:** 1 Research Center of Transport Proteins for Medical Innovation, Department of Physiology, Mahidol University, Bangkok 10400, Thailandvaranuj.cha@mahidol.ac.th (V.C.); 2 Excellent Center for Drug Discovery, Mahidol University, Bangkok 10400, Thailand

**Keywords:** Nuclear receptor, renal excretion, kidney, drug transporters, bile acids

## Abstract

Farnesoid X receptor (FXR) is a ligand-activated transcription factor highly expressed in the liver and kidneys. Activation of FXR decreases organic cation transporter (OCT) 1-mediated clearance of organic cation compounds in hepatocytes. The present study investigated FXR regulation of renal clearance of organic cations by OCT2 modulation and multidrug and toxin extrusion proteins (MATEs). The role of FXR in OCT2 and MATEs functions was investigated by monitoring the flux of ^3^H–MPP^+^, a substrate of OCT2 and MATEs. FXR agonists chenodeoxycholic acid (CDCA) and GW4064 stimulated OCT2-mediated ^3^H–MPP^+^ uptake in human renal proximal tubular cells (RPTEC/TERT1 cells) and OCT2-CHO-K1 cells. The stimulatory effect of CDCA (20 µM) was abolished by an FXR antagonist, Z-guggulsterone, indicating an FXR-dependent mechanism. CDCA increased OCT2 transport activity via an increased maximal transport rate of MPP^+^. Additionally, 24 h CDCA treatment increased MATEs-mediated ^3^H-MPP^+^ uptake. Moreover, CDCA treatment increased the expression of OCT2, MATE1, and MATE2-K mRNA compared with that of the control. OCT2 protein expression was also increased following CDCA treatment. FXR activation stimulates renal OCT2- and MATE1/2-K-mediated cation transports in proximal tubules, demonstrating that FXR plays a role in the regulation of OCT2 and MATEs in renal proximal tubular cells.

## 1. Introduction

The kidney is largely responsible for the elimination of metabolic waste products, therapeutic drugs, and xenobiotics, which contain organic cations (OCs) and anions (OAs) [1]. The secretion of OCs takes place in renal proximal tubules. This process requires the uptake of OCs from the blood into renal proximal tubular cells and subsequent elimination of these compounds into the tubular lumen across the luminal membrane. Three members of the organic cation transporters (OCTs), including OCT1, OCT2, and OCT3, are characterized [2]. Human OCT1 and OCT2 are highly expressed in liver and kidney, respectively, whereas OCT3 is ubiquitously expressed at a low level in multiple tissues [3]. OCs are transported into renal proximal tubular cells via the organic cation transporter (OCT) 2, a predominant OCT expressed in the basolateral membrane of human renal proximal tubular cells [4,5]. OCT2-mediated uptake of OCs is governed by an inside-negative membrane potential [4,6]. After uptake, OCs are then effluxed to the tubular lumen by several apical membrane transporters such as multidrug and toxin extrusion proteins (MATEs). Two MATE isoforms, MATE1 and MATE2-K, are expressed in renal proximal tubular cells [7,8]. Several endogenous compounds and therapeutic cationic drugs are eliminated via renal excretion that have been identified as substrates of both OCT2 and MATEs such as creatinine and metformin [5,9,10,11,12,13].

Altered expression or function of these transporters can affect organic cation drug renal secretion and subsequently alter their pharmacokinetics and efficacies [14,15]. Studies have reported nuclear receptor-mediated regulation of OCT2 and MATEs function. Specifically, the activation of pregnane X receptor (PXR) and androgen receptor increased OCT2 expression [16,17], whereas liver X receptor (LXR) activation decreased OCT2 expression and function [18]. In addition, kidney-specific multidrug and toxin extrusion proteins (MATE2K) expression was up-regulated upon the activation of Nrf2 pathway signaling [19]. Previous studies have shown that farnesoid X receptor (FXR), a ligand-activated transcriptional factor, is highly expressed in liver, kidney, intestine, and adrenal gland tissue [20]. FXR regulates several membrane transporters and channels, including the bile salt export pump (BSEP) [21], multidrug resistance-associated protein 2 (MRP2) [22], organic solute transporter OSTα/β [23,24], aquaporin 2(AQP2) [25], and MATE1 [26].

Previous studies showed that cholestasis resulted in a down-regu lation of OCT1 and impairment of hepatic-mediated OCT1 substrate uptake [27,28]. These processes might be controlled by the activation of FXR by bile acids, such as cholic acid and chenodeoxycholic acid (CDCA). These bile acids have been reported to act as endogenous ligands for FXR [29] and are increased in hepatic disease [30]. Down-regulation of OCT1 regarded as adaptive responses to cholestasis and may serve to diminish the hepatic accumulation of cationic substrate during liver injury [31]. Since OCT1 plays a role in the hepatic uptake-mediated biotransformation and the excretion of endogenous compounds and cationic drugs, decreases in OCT1 function may result in increased cationic plasma concentration. We hypothesize that other cation transporters expressed in the kidney, such as OCT2 and MATEs, may be critically important for cationic substrate elimination in hepatic disease. Here, we investigated the effect of FXR activation on renal OCT2 and MATEs function in renal proximal tubular cells.

## 2. Results

### 2.1. FXR Agonists Stimulate OCT2-Mediated ^3^H-MPP^+^ Uptake

**Figure 1:**
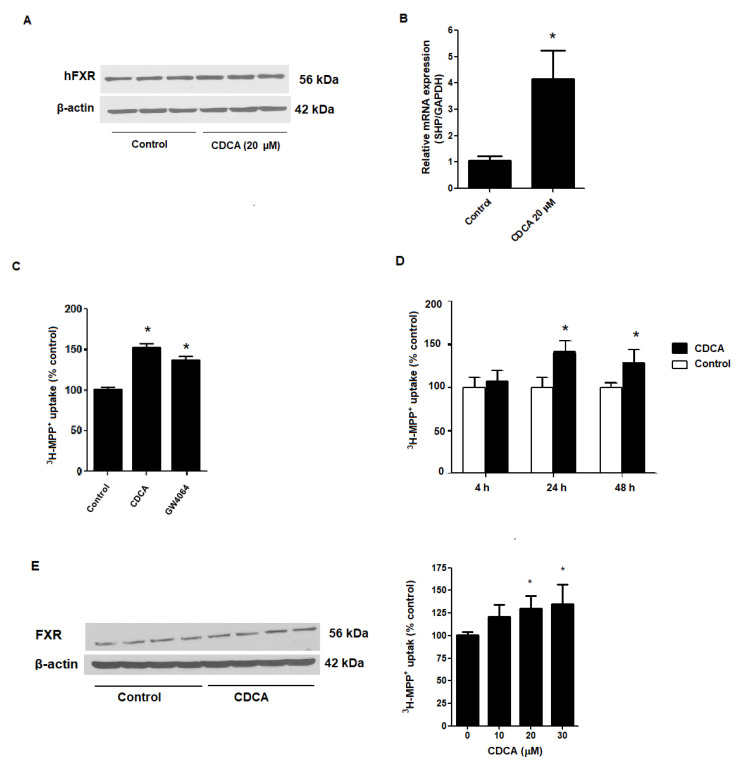
Effect of FXR agonists on OCT2-mediated ^3^H–MPP^+^ uptake. (**A**) protein expression of FXR and (**B**) mRNA expression of SHP in RPTEC/TERT1 cells following treating the cells with vehicle or 20 µM CDCA for 24 h; (**C**) effect of 20 µM CDCA and 5 µM GW4064 on ^3^H-MPP^+^ uptake; (**D**) Time-response effect of 20 µM CDCA; (**E**) FXR expression and ^3^H-MPP^+^ uptake in OCT2-CHO-K1 cells following incubation with 20 µM CDCA for 24 h. Data are expressed as a mean percentage of control (mean ± S.D.) from 3 independent experiments. *Significantly different from control (*p* < 0.05).

To be certain that RPTEC/TERT1 cells are suitable cell model for investigating the role of FXR, we first tested whether the RPTEC/TERT1 cells express FXR by examination protein expression via Western blot analysis. As shown in Figure 1, we confirmed FXR protein expression in RPTEC/TERT1 cells. Relative to untreated cells, mRNA expression of a small heterodimer partner (SHP; a target gene of FRX activation [32,33]) was significantly increased following treatment with 20 µM CDCA for 24 h (Figure 1A,B). Next, we tested the effects of CDCA and GW4064, a potent synthetic agonists of FXR, on OCT2-mediated ^3^H-MPP^+^ uptake. As shown in Figure 1C, 24 h incubation with 20 µM CDCA and 5 µM GW4060 significantly stimulated OCT2-mediated ^3^H-MPP^+^ cellular uptake. While 20 µM CDCA stimulated uptake after 24 h, an extended incubation time did not lead to any further increase in OCT2-mediated ^3^H-MPP^+^ uptake (Figure 1D). In addition, we confirmed the effect of CDCA on OCT2-mediated ^3^H-MPP^+^ uptake in CHO-K1 cells expressing OCT2. Specifically, 24 h incubation with 20 and 30 µM CDCA significantly promoted ^3^H-MPP^+^ uptake in CHO-K1 cells (Figure 1E).

### 2.2. Stimulatory Effects of FXR Agonists Require FXR Activation

**Figure 2:**
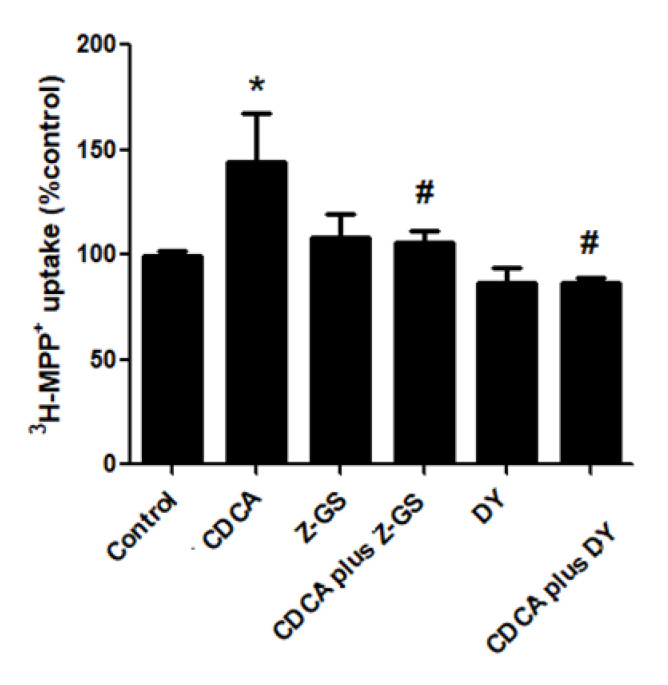
Effect of FXR activation on OCT2-mediated ^3^H–MPP^+^ uptake. RPTEC/TERT1 cells were treated with vehicle, 20 µM CDCA, FXR antagonists (10 µM Z-guggulsterone (Z-GS) or 10 µM DY268), and CDCA plus FXR antagonist for 24 h. The results are shown as mean ± S.D. of % control form 4 experiments. *Significantly different from control (*p* < 0.05) and # p < 0.05 compared with CDCA-treated cells.

To determine whether the CDCA stimulation of ^3^H-MPP^+^ uptake is directly caused by FXR activation, we examined how FXR antagonists, Z-guggulsterone and DY268, affect CDCA-induced stimulation of ^3^H-MPP^+^ uptake. As shown in Figure 2, exposure to 10 µM Z-guggulsterone or DY268 had no significant effect on ^3^H-MPP^+^ uptake. CDCA-mediated uptake stimulation was attenuated by coincubation with Z-guggulsterone or DY268. These data indicate that CDCA uptake stimulation requires FXR activation.

### 2.3. Kinetic Study on FXR Activation on OCT2-mediated ^3^H-MPP^+^ Uptake

**Figure 3:**
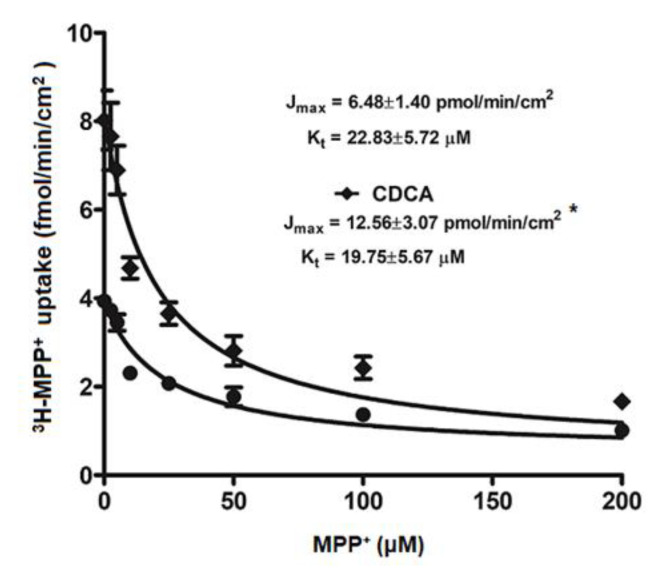
Kinetic study of OCT2-mediated ^3^H-MPP^+^ uptake in RPTEC/TERT1 cells. RPTEC/TERT1 cells were treated with vehicle or 20 µM CDCA for 24 h. ^3^H-MPP^+^ uptake was determined in the presence of unlabeled MPP^+^ at 0–200 µM. The Jmax, and Kt values are reported as mean ± S.D. (*n* = 3). *Significantly different from control (*p* < 0.05).

To investigate how FXR activation stimulates ^3^H-MPP^+^ uptake, we evaluated the kinetic parameters Kt and Jmax that reflect an affinity and functional membrane expression of OCT2, respectively. As shown in Figure 3, 24 h treatment with 20 μM CDCA in RPTEC/TERT1 cells significantly increased the Jmax from 6.48 ± 1.4 to 12.56 ± 3.1 pmol/min/cm^2^ with no significant effect on Kt (22.83 ± 5.7 vs 19.75 ± 5.67 µM).

### 2.4. FXR Activation Increases mRNA and Protein Expression of OCT2 

**Figure 4:**
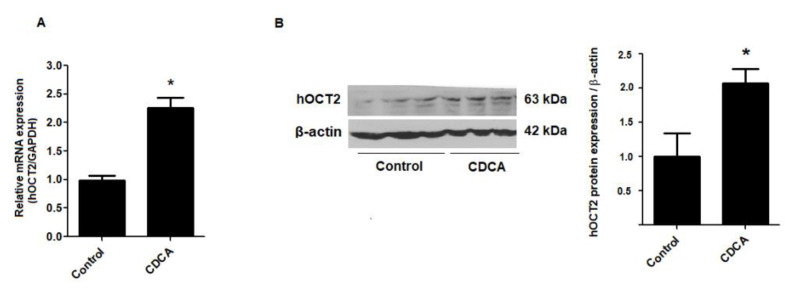
Effect of CDCA on expression of hOCT2. (**A**) mRNA expression of hOCT2 after treating with vehicle or 20 µM CDCA for 24 h. (**B**) Representative blots and the densitometry quantification of hOCT2 expression normalized by β-actin. The data are shown as mean ± S.D. from three independent experiments. * *p* < 0.05 compared with vehicle-treated group.

To determine whether FXR activation affects OCT2 expression, RPTEC/TERT1 cells treated with vehicle or 20 μM CDCA for 24 h were probed for OCT2 mRNA and protein expression. Treatment of RPTEC/TERT1 cells with 20 μM CDCA significantly increased OCT2 mRNA expression compared with vehicle treatment. In addition, 20 μM CDCA treatment led to an increase in OCT2 protein expression as shown in Figure 4.

### 2.5. FXR Activation Increases Function and Expression of MATEs 

**Figure 5:**
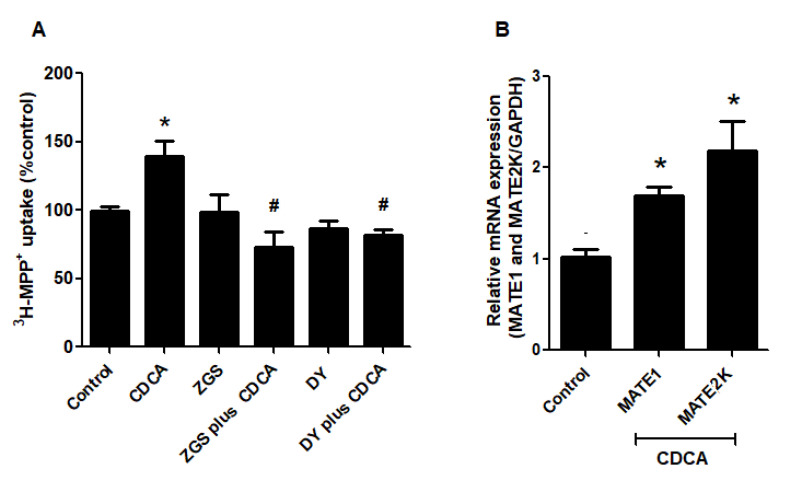
The effect of FXR activation on MATEs-mediated ^3^H–MPP^+^ uptake and MATEs expression in RPTEC/TERT1 cells. (**A**) MATEs-mediated ^3^H–MPP^+^ uptake; treated with vehicle, 20 µM CDCA, FXR antagonists (10 µM Z-guggulsterone (Z-GS) or 10 µM DY268 (DY)), and CDCA plus FXR antagonists. (**B**) mRNA expression of MATE1 and MATE2K. The data are shown as mean ± S.D. (*n* = 3). * *p* < 0.05 compared with control and # *p* < 0.05 compared with CDCA-treated cells.

Regulation of MATEs transport function by FXR activation was determined in RPTEC/TERT1 cells. These transporters function as organic cation/H^+^ exchangers and are driven by a proton-gradient. Therefore, to test MATEs-mediated ^3^H–MPP^+^ uptake, we preincubated RPTEC/TERT1 cells with a K^+^ based buffer containing ammonium chloride to generate intracellular acidification before transport measurement. Consequently, 20 µM CDCA treatment for 24 h significantly increased MATEs-mediated ^3^H-MPP^+^ compared with the vehicle-treated cells. CDCA stimulation was significantly inhibited by Z-guggulsterone and DY268 (Figure 5A). Next, we tested whether the observed correlation between FXR activation and MATEs transport function was a result of MATEs mRNA up-regulation. Using RPTEC/TERT1 cells, MATE1 and MATE2K mRNA expression were analyzed following treatment with 20 µM CDCA for 24 h. CDCA significantly increased both MATE1 and MATE2K mRNA expression (Figure 5B).

### 2.6. FXR Activation Stimulates Transepithelial Transport of ^3^H-MPP^+^

**Figure 6:**
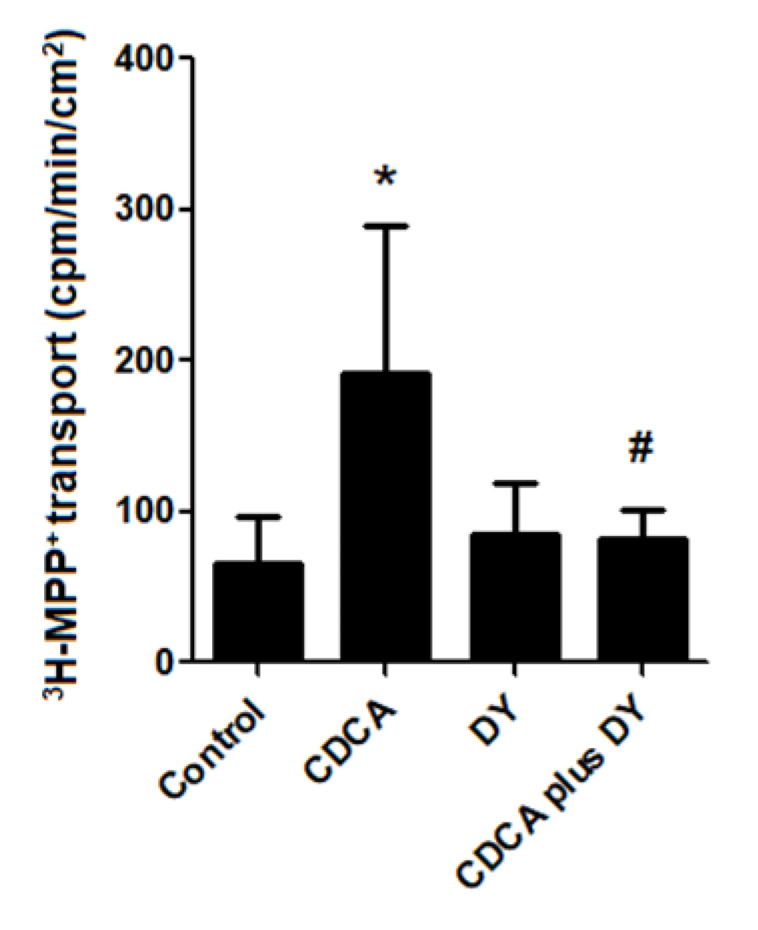
Effect of CDCA on transcellular transport of ^3^H-MPP^+^ in polarized cell monolayer. Polarized RPTEC/TERT1 cell monolayers were treated with vehicle, 20 µM CDCA, 10 µM DY268 (DY), and CDCA plus DY for 24 h. The values of basolateral to apical transport of ^3^H-MPP^+^ are expressed as mean ± S.D. of cpm/min/cm^2^ form 3 experiments. Data from each experiment is obtained from 3 inserts. * *p* < 0.05 compared with vehicle-treated group and ^#^ *p* < 0.05 compared with CDCA-treated cells.

To determine the relationship between FXR activation and transcellular transport of OCs, we examined the effect of FXR activation on basolateral-apical transport of ^3^H-MPP^+^. As such, cell monolayers were incubated with vehicle control, 20 µM CDCA, 10 µM DY268, and 20 µM CDCA plus 10 µM DY268, and transepithelial transport of ^3^H-MPP^+^ was measured after 24 h. As shown in Figure 6, transcellular translocation of ^3^H-MPP^+^ from the basolateral to the apical chamber was significantly higher in the CDCA-treated cell monolayer compared with the vehicle-treated cells. Importantly, the stimulatory effect of CDCA was abolished by co-treatment with DY268.

### 2.7. Pathological Concentration of Bile Acid Stimulates Renal OCT2 and MATEs

**Figure 7:**
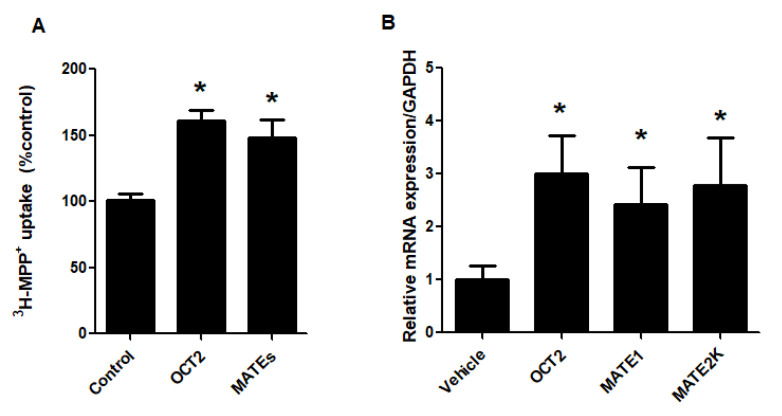
Effect of high concentration of CDCA on function and expression of OCT2 and MATEs. RPTEC/TERT1 cells were treated with vehicle or 80 µM CDCA for 24 h followed by measurements of (**A**) OCT2- and MATEs-mediated ^3^H-MPP^+^ uptake and (**B**) mRNA expression of OCT2, MATE1, and MATE2K. The data are expressed as mean ± S.D. from 3 independent experiments. * *p* < 0.05 compared with vehicle-treated group.

Previous studies have reported an increased concentration of unconjugated bile acids in liver diseases [30]. Therefore, we investigated the correlation between high unconjugated bile acid concentration and the stimulation of renal OCs transport. Cell monolayers were incubated with CDCA at 80 µM for 24 h followed by measurement of OCT2- and MATEs-mediated ^3^H-MPP^+^ transport. As shown in Figure 7, treatment a pathological concentration of CDCA significantly stimulated both OCT2- and MATEs-mediated ^3^H-MPP^+^ compared with vehicle-treated cells. The stimulatory effect of 80 µM CDCA on ^3^H-MPP^+^ uptake was correlated with an increase in mRNA expression of OCT2, MATE1, and MATE2K.

## 3. Discussion

Cationic transporters play a crucial role in the renal clearance of cationic endogenous and xenobiotic compounds [1,34]. Therefore, the altered expression and/or function of these transporters could affect the total profile excretion of these compounds. Previous reports showed that FXR activation regulates several hepatic transporters in different manners including down-regulation of OCT1 protein expression [27] or up-regulation of MATE1 protein expression [26]. The present study revealed that FXR activation regulates renal OCT2 and MATEs expression and function in the human proximal tubular cell line RPTEC/TERT1. Importantly, RPTEC/TERT1 cells express OCT2, MATE1, and MATE2K and represent an important in vitro model for studying renal transport [35]. Although the expression of FXR is present in the proximal tubular cells, we verified whether RPTEC/TERT1 cell line was suitable as a study model of FXR function. For this study, we initially confirmed that this cell line could be used for studying FXR activation by showing that FXR is expressed and activated by FXR agonist. Of note, we showed that FXR agonists increased OCT2-mediated ^3^H-MPP^+^ uptake in RPTEC/TERT1 cells. Importantly, the effect of CDCA on OCT2-mediated ^3^H-MPP^+^ uptake was not observed until after 24 h of incubation time; indicating that the FXR agonist has a slow mode of action on OCT2. Moreover, we showed that CDCA modulation of OCT2 is dependent upon FXR activation, as evidenced by our result showing that inhibition of FXR by pharmacological antagonists, guggulsterone and DY268 [36,37], attenuated the CDCA-mediated stimulation. Since RPTEC/TERT1 cells express both OCT2 and OCT3 [35], total ^3^H-MPP^+^ uptake into RPTEC/TERT1 cells could be mediated by either. However, we proved here that CDCA also stimulates OCT2 transport function in CHO-K1 cells expressing only OCT2. Taken together, these data imply that FXR activation by CDCA stimulates OCT2-mediated ^3^H-MPP^+^ uptake into RPTEC/TERT1 cells. However, we cannot rule out possible stimulatory effects of FXR activation on OCT3 in RPTEC/TERT1 cells. How FXR activation affects OCT3 should be further investigated in cells expressing OCT3 alone. 

The stimulatory effect of FXR activation on OCT2 transport might via increase in either the functional number of transporters and/or transporter affinity with its substrate. Using kinetic data, we revealed that CDCA treatment increase in the Jmax of OCT2-mediated transport function. This result is indicative of an increase in the number of transporters at the membrane surface. FXR activation regulates several renal transporters and channel such as the organic solute transporters α and β (OSTα and OSTβ) and AQP2 by increase in mRNA and protein expressions [24,25], we investigated whether increased expression of OCT2 mediated the stimulatory effect of CDCA. We found that CDCA increased OCT2 mRNA and protein expression in RPTEC/TERT1 cells. These results indicate that FXR activation increases ^3^H-MPP^+^ uptake into renal proximal tubular cells via the up-regulation of OCT2 mRNA and protein. Although previous studies have demonstrated that FXR modulates transporter and channel gene expression [24,25], we did not explore FXR direct binding and the up-regulation of OCT2 gene expression. FXR direct modulation of OCT2 expression needs to be determined in future studies. 

Renal secretion of OCs requires both basolateral uptake and apical efflux. Therefore, we also investigated the correlation of FXR activation with the transporters expressed at apical membrane mediating secretion of OCs including MATE1 and MATE2K [8]. Our data demonstrated that CDCA stimulated the transport function of MATEs. Since FXR antagonists abolish this effect, we showed that the CDCA-mediated stimulation was controlled directly through FXR. The correlative relationship between FXR activation and OCT2/MATEs transport function was confirmed in basolateral to apical experiments. We demonstrated that FXR activation drove the flux of ^3^H-MPP^+^ from the basolateral chamber to the apical chamber. These results indicate that FXR activation stimulates OCT2/MATEs-mediated OC secretion in renal proximal tubular cells. Since RPTEC/TERT1 cells express both MATE1 and MATE2K, it is unclear what isoform FXR stimulated. Furthermore, we found that the stimulatory effect of FXR activation on ^3^H-MPP^+^ transport correlated well with the up-regulation of MATE1 and MATE2K mRNA. These results provide evidence that FXR activation stimulates OC secretion via up-regulation of both MATE1 and MATE2K. 

There is increasing evidence that FXR is a critical regulatory factor in renal physiology and pathophysiology [38]. Previous studies have shown that pathological conditions related to hepatic injuries, such as ischemia/reperfusion and cholestasis, result in an up-regulation of the efflux transporter MATE1 and down-regulation of the uptake transporter OCT1 [27]. Dysregulation of these transporters can lead to the reduction of accumulated cationic compounds in hepatocytes [27]. Altered hepatic function could result in an increased plasma concentration of cations. Pathological conditions in the liver, such as acute hepatitis and obstructive jaundice, significantly increase the total serum concentrations of unconjugated bile acid including cholic acid, deoxycholic acid, and chenodeoxycholic acid (endogenous FXR agonists) up to 100 µM [29,30]. To model the consequences of pathologically high bile acid concentrations, we sought to correlate the modulation of bile acid concentration with renal OCT2, MATE1, and MATE2K expressions and functions. Our results revealed that high CDCA concentrations increase OCT2 and MATEs mRNA expression and modulate their function. These findings correlate with a previous study, which found that renal clearance of OCs in acute hepatic injury was increased due to high protein expression of OCT2 in the renal cortex [39]. Taken together, these data imply that renal FXR activation may be an adaptive response for the clearance of excess plasma OCs during hepatic clearance impairment.

## 4. Materials and Methods

### 4.1. Chemicals

N-methyl-^3^H-4-phenylpyridinium acetate (^3^H-MPP^+^; 80 Ci/mmol) was purchased from American Radio Labeled Chemical Inc. (St. Louis, MO, USA). DY268 was purchased from Trocris (Thai Can Biotech, Bangkok, Thailand). Dulbecco’s modified Eagle’s medium (DMEM), Ham’s F-12 nutrient mix (1:1), and TRIzol reagent, products of Invitrogen, were purchased from Gibthai (Bangkok, Thailand). iScrip cDNA Synthesis Kit and Luna Universal qPCR mastermix were obtained from Bio-Rad Thailand (Bangkok, Thailand), GW4064 (synthetic FXR agonist). Chenodeoxycholic acid (CDCA), Z-guggulsterone (FXR antagonist), tetrapentylammonium (TPeA), methyl-4-phenylpyridinium (MPP^+^), and human OCT2 (HPA008567) antibody were purchased from Sigma-Aldrich (Bangkok, Thailand). Antibodies against FXR and β-actin were purchased from Merck Millipore (Bangkok, Thailand). Other chemicals used were of analytical grade from commercial sources.

### 4.2. Cell Cultures

RPTEC/TERT1 cells, an immortalized renal proximal tubular cell line expressing several drug transporters [35,40], was obtained from American Type Culture Collection (ATCC) and cultured in a mixture of DMEM and Ham’s F-12 (1:1) supplemented with 10 ng/mL human epithelial growth factor, 5 μg/mL insulin, 5 μg/mL human transferrin, 5 ng/mL sodium selenite, 36 ng/mL hydrocortisone, 100 U/mL penicillin, and 100 μg/mL streptomycin. CHO-K1 cells expressing rbOCT2 were kindly gifted from Professor Stephen Wright, University of Arizona. These cells were maintained in Ham’s F12 media supplemented with 10% FBS, 100 U/mL penicillin, and 100 μg/mL streptomycin, and 1% G418. All cells were cultured in a humidified incubator with 5% CO_2_/95% air at 37 °C.

### 4.3. Measurement of OCT2 Transport Function

OCT2-mediated in ^3^H-MPP^+^ uptake in RPTEC/TERT1 cells was measured as previously described [18]. Briefly, RPTEC/TERT1 cell monolayers were washed twice with 1 mL of warm buffer pH 7.40 (NaCl 135 mM, KCl 5 mM, HEPES 13 mM, CaCl_2_.2H_2_O 2.5 mM, MgCl_2_ 1.2 mM, MgSO4.7H_2_O 0.8 mM and D-glucose 28 mM) and incubated for further 15 min. The cell monolayers were incubated with buffer containing ^3^H-MPP^+^for 5 min. The transport was stopped by three times washing with ice-cold buffer containing 100 μM unlabeled MPP^+^. Cells were then lysed by adding 200 μL of 0.4 N NaOH in 10% SDS and left overnight. To neutralize the sample pH, 80 µL of 1 N HCl was added into each well. Accumulation of labeled MPP^+^ was determined with a liquid scintillation and calculated as mole/min/cm^2^ of the confluent monolayer surface.

### 4.4. Measurement of MATEs Transport Function

Measurement of MATEs-mediated ^3^H-MPP^+^ transport in RPTEC/TERT1 cells was performed as described by previous study [41]. Briefly, the cell monolayers were washed twice with 1 mL of warm K^+^-based buffer (pH 7.4; KCl 130 mM, MgSO_4_.7H_2_O 1.2 mM, CaCl_2_.2H_2_O 1 mM, K_2_HPO_4_ 2 mM, HEPES 20 mM, and D-glucose 5 mM) and were incubated for 15 min at 37 °C. To manipulate the intracellular acidification, the cell monolayers were further incubated with K^+^-based buffer containing NH_4_Cl 30 mM for 20 min at 37 °C [42,43]. Then, the cell monolayers were incubated with 200 μl of K^+^ based-buffer (pH 8.0) containing ^3^H-MPP^+^ for 10 min. After incubation, the cell monolayers were washed three times with ice-cold buffer containing unlabeled MPP^+^ 100 μM to stop transport activity. The cells were lysed by 0.4 N NaOH in 10% SDS and cellular accumulation of ^3^H-MPP^+^ was measured and calculated as fmol/min/cm^2^ of the confluent monolayer surface. 

### 4.5. Basolateral to Apical Transport of ^3^H-MPP^+^

RPTEC/TERT1 cells were cultured in in Transwell 12-well cultures (0.4 µm pore size; Corning Life Science, Corning, NY, USA) for 21 days. Basolateral and apical chambers were filled with 1 and 0.5 mL of media, respectively. Cell monolayer integrity was assessed using transepithelial electrical resistance (TEER). We selected the cell monolayers that achieved TEER values > 100 Ω·cm^2^. On the day of experiment, the culture medium was withdrawn, and replaced with warm transport buffer and incubated with warm buffer for 30 min at 37 °C. Basolateral chamber was added with ^3^H-MPP^+^ for 30 min followed by sample collection (0.2 mL) from the apical chamber to determine ^3^H-MPP^+^ transepithelial transport. Transporter-mediated ^3^H-MPP^+^ transport was calculated by subtraction the total basolateral to apical transport of ^3^H-MPP^+^ with the transport of ^3^H-MPP^+^ in the presence of TPeA 100 µM, an inhibitor of OCTs.

### 4.6. Kinetic Analysis of OCT2-mediated ^3^H-MPP^+^ Uptake

The evaluation of OCT2 transport kinetics was performed as described previously [18]. RPTEC/TERT1 cell monolayers were incubated with transport buffer containing ^3^H-MPP^+^ 10 nM in the presence of various concentrations of unlabeled MPP^+^. ^3^H-MPP^+^ uptake was calculated as mole/min/cm^2^ of the confluent monolayer surface. This was followed by the calculation of kinetic parameters including a maximum transport rate of MPP^+^ (Jmax) and the concentration of unlabeled MPP^+^ that resulted in half-maximal transport (Kt) using the Michaelis–Menten equation of competitive interaction between labeled and unlabeled MPP^+^ [44].

### 4.7. Real-Time PCR

Total RNA from RPTEC/TERT1 cells was extracted using TRIzol reagent (Invitrogen, Bangkok, Thailand). Synthesis of cDNA was performed using iScript cDNA Synthesis Kit (Bio-Rad, Bangkok, Thailand). A Luna Universal qPCR mastermix was then utilized for PCR amplification (Bio-Rad, Bangkok, Thailand). The primers used in this study are shown in Table 1.

The cycle threshold (CT) values were obtained from ABI Prism 7500 Sequence Detection System (Applied Biosystems (Thailand), Bangkok, Thailand), and the relative expression levels of mRNA were determined by the 2^−ΔΔCt^ method [45].

### 4.8. Western Blot Analysis

Proteins of RPTEC/TERT1 cells were separated by 10% SDS-polyacrylamide gel electrophoresis and subsequently transferred to a nitrocellulose membrane. Membranes were blocked with 5% non-fat dry milk for 2 h at room temperature and then blotted with primary antibodies for overnight at 4 °C. After that, the membranes were washed four times with Tris-buffered saline (TBST) for 10 min each. Subsequently, the membranes were incubated with horseradish peroxidase (HRP)-conjugated secondary antibody (Merck Millipore, Bangkok, Thailand) for 1 h. Proteins were detected and quantified by using an enhanced chemiluminescence (ECL) detection kit (Merck Millipore, Bangkok, Thailand) and the Gel and Graph Digitizing System (Uvitec, Cambridge, UK), respectively.

### 4.9. Statistical Analysis

Data are presented as mean and standard deviation (mean ± S.D.). Data of the kinetic study were analyzed by using unpaired student *t*-tests whereas other data were analyzed by using one-way analysis of variance (one-way ANOVA) tests with a post hoc Newman–Keuls test. The significant difference between each group of data was considered when *p* < 0.05.

## 5. Conclusions

We have demonstrated that FXR activation stimulates OC secretion in human renal proximal tubular cells. Moreover, the stimulatory effect of FXR on renal OC secretion may be mediated by the increase in OCT2/ MATEs-mediated OC transport. This effect is likely caused by enhanced OCT2 and MATE1/2K expression. Taken together, this study enhances our understanding of the role FXR may play in the regulation of renal OCT2- and MATEs-mediated renal OCs excretion.

## Figures and Tables

**Table 1:** Primers (forward/reverse) for real-time PCR.

Target	Forward Primer (5′-3′)	Reverse Primer (3′-5′)
hOCT2	5-AGTCTGCCTGGTCAATGCT-3	5-AGGAATGGCGTGATGATGC-3
hMATE1	5-TGCTCCTGGGGGTCTTCTTA-3	5-GTGGGCCTGTGAATTGTGTG-3
hMATE2-K	5-TTGCACAGACCGTCTTCCTC-3	5-TGAGGAAGCTCCCGATCTCA-3
hSHP	5-GGCTTCAATGCTGTCTGGAGT-3	5-CTGGCACATCGGGGTTGAAGA-3
hGAPDH	5-CAAGCTCATTTCCTGGTATGAC-3	5-GTGTGGTGGGGGACTGAGTGTGG-3
